# Supplementary material for: Salmonella effector kinase SteC is activated by phosphorylation at Serine 379
Source: PLoS Pathog. 2026 Jul 16;22(7):e1014424. doi: 10.1371/journal.ppat.1014424 (PMC13395416; doi:10.1371/journal.ppat.1014424)
Supplement: S1 Fig — (DOCX) [file ppat.1014424.s001.docx]

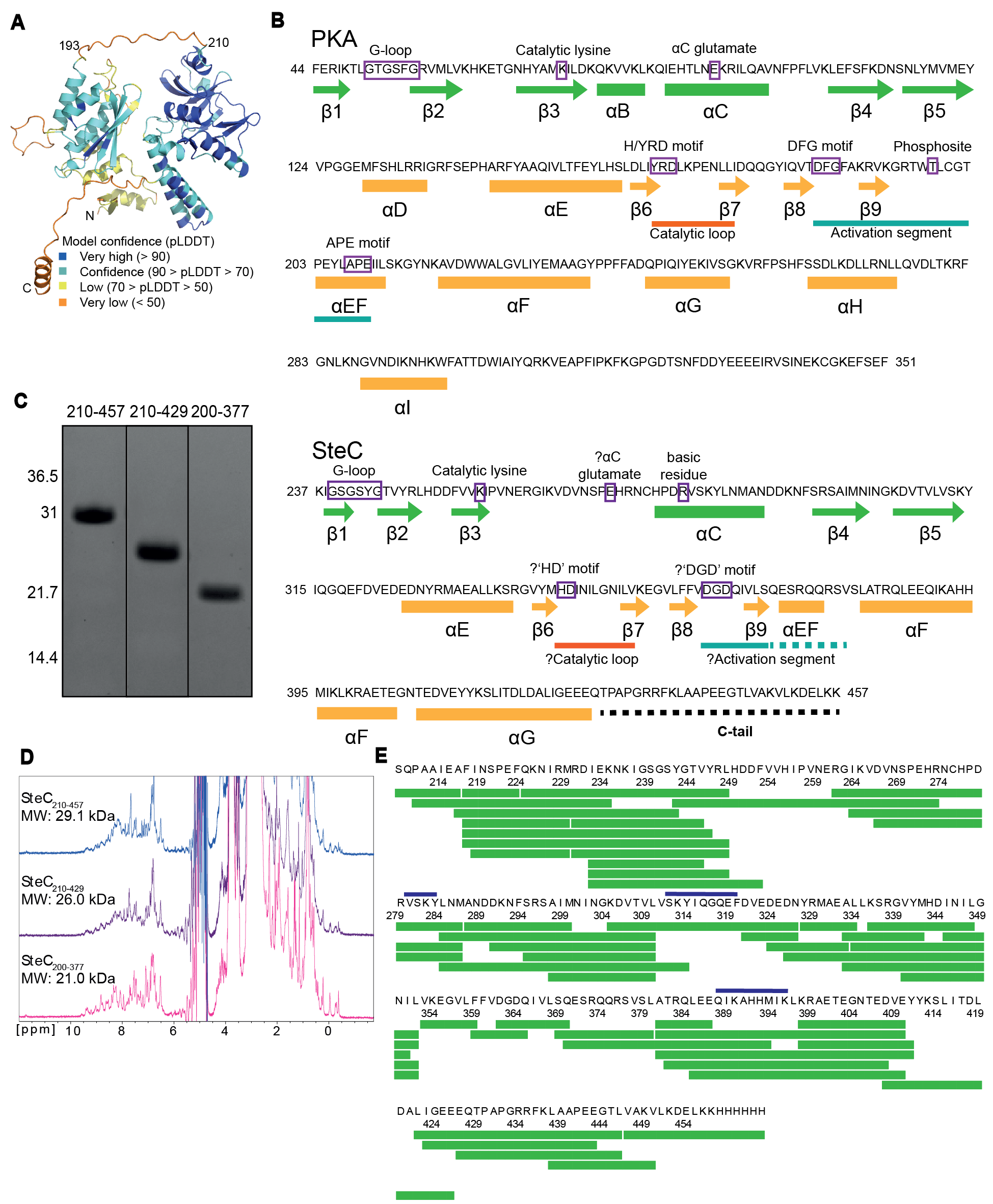


#### **S1** **Fig: Predicted and experimentally derived features of the structure of SteC**

1. AlphaFold2 prediction of SteC, with N-terminal regulatory domain shown left, kinase domain right, and the C-tail crossing over at the front. Amino acids are coloured with AlphaFold2-generated predicted local distance difference test (pLDDT) score as per key.
2. Top: PKA key sequence motifs (purple) are highlighted alongside secondary structure elements: α-helices and β-sheets comprising the N lobe (green) and C lobe (orange); the catalytic loop, activation segment and activation loop are indicated. Adapted from Sheetz and Lemmon (2022)^26^. Bottom: SteC with secondary structure elements as per AF prediction in N lobe (green) and C lobe (orange); hypothetical key motifs, and possible catalytic loop, activation segment, activation loop and C-tail are indicated in purple, with dotted lines referring to non-canonical features.
3. SDS-PAGE analysis of SteC encompassing amino acids 210-457, 210-429 and 200-377 expressed and purified from *E. coli*.
4. 1D ^1^H-NMR spectra of SteC K256H mutants of 210-457, 210-429 and 200-377 expressed in *E. coli* collected at 25 °C on a Bruker AVANCE spectrometer operating at 800 MHz.
5. Peptide coverage of Hydrogen deuterium exchange experiments of SteC 210-457 K256H expressed in *E. coli*. Total 53 peptides, 100% coverage, 3.80 redundancy. Regions significantly protected in the presence of the FMNL1 peptide are highlighted in blue above. Regions of SteC demonstrating protection in the presence of the peptide (at least 0.45 difference in all peptides covering this region) are highlighted on the AF predicted structure in blue in **Fig 1C**.
