# Supplementary material for: Salmonella effector kinase SteC is activated by phosphorylation at Serine 379
Source: PLoS Pathog. 2026 Jul 16;22(7):e1014424. doi: 10.1371/journal.ppat.1014424 (PMC13395416; doi:10.1371/journal.ppat.1014424)
Supplement: S2 Fig — (DOCX) [file ppat.1014424.s002.docx]

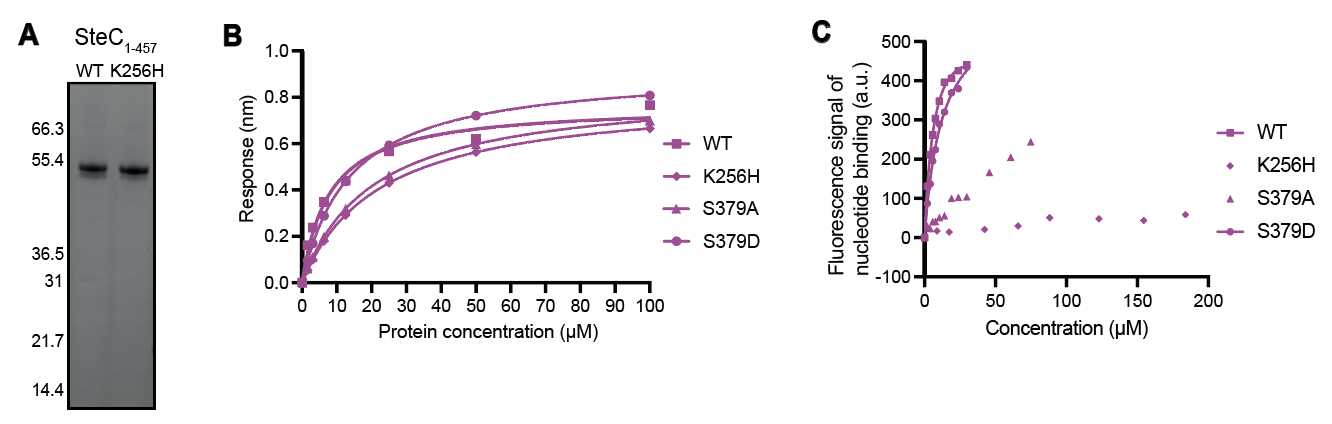


#### **S2 Fig: Kinase activity and substrate binding of SteC**

1. Coomassie stained SDS-PAGE analysis SteC_1-457_ WT and K256H expressed in Sf9 cells.
2. Analysis of the interaction of SteC_210-429_ WT and mutants expressed in *E. coli* with an FMNL1 peptide by Biolayer Interferometry. The biotinylated peptide was loaded onto Streptavidin sensors. Fitted curves are shown as purple lines.
3. Fluorescence titrations of 500 nM mant-AMPPNP with SteC_210-429_ WT and mutants expressed in *E. coli*, as in **Fig 2G**. Higher concentrations of SteC_K256H_ and SteC_S379A_ were used. Fitted curves are shown as purple lines.
