# Supplementary material for: Salmonella effector kinase SteC is activated by phosphorylation at Serine 379
Source: PLoS Pathog. 2026 Jul 16;22(7):e1014424. doi: 10.1371/journal.ppat.1014424 (PMC13395416; doi:10.1371/journal.ppat.1014424)
Supplement: S3 Fig — (DOCX) [file ppat.1014424.s003.docx]

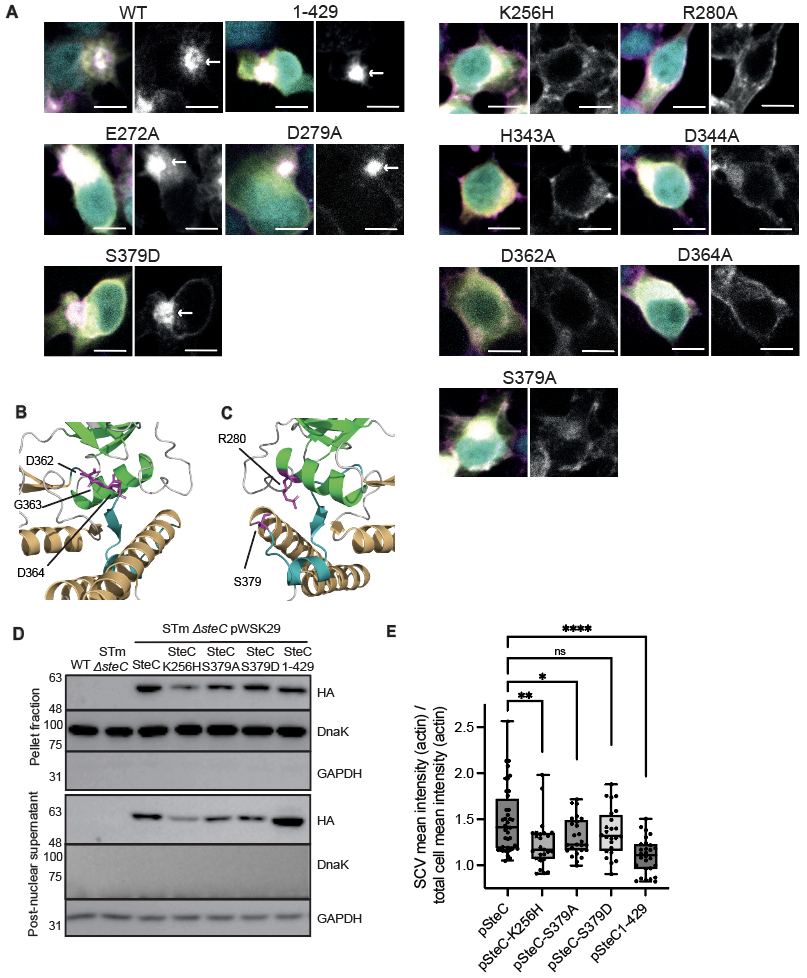


#### **S3 Fig: SteC in infected and transfected mammalian cells**

1. HEK 293ETs were transfected with the indicated GFP-SteC variants and following fixation, were stained with 4’6-diamidino-2-phenylindole (DAPI) and phalloidin AF647 prior to imaging. Representative merged images with DAPI (cyan), phalloidin (magenta) and GFP (yellow) and a grayscale for the phalloidin channel (right) are shown for each condition. Representative cells were chosen randomly. Scale bar represents 5 μm. White arrows denote actin polymerisation within the transfected cell.
2. As for **Fig 3A**, but with D362, G363 and D364 shown in purple sticks.
3. As for **Fig 3A**, but rotated 180° in the y axis, with S379 and R280 shown in purple sticks, indicating their proximity (4 Å apart).
4. 3T3 cells were infected with *Salmonella* WT, *ΔsteC* and *ΔsteC psteC* strains. Eight hours post invasion, cells were lysed and pellet and post-nuclear fractions analysed by immunoblotting with antibodies against HA (SteC), DnaK and GAPDH. Data representative of 2 independent biological repeats.
5. The ratio of mean intensity of phalloidin signal around *Salmonella* microcolonies compared to within the cell mask for each strain. Each data point represents a field of view, each box represents the mean and interquartile range (IQR), while whiskers extend to 1.5xIQR. Independent two-sided T-test with Welch correction was used to determine p-values. * – <0.05, ** – <0.01, *** – <0.001.
