# Supplementary material for: Salmonella effector kinase SteC is activated by phosphorylation at Serine 379
Source: PLoS Pathog. 2026 Jul 16;22(7):e1014424. doi: 10.1371/journal.ppat.1014424 (PMC13395416; doi:10.1371/journal.ppat.1014424)
Supplement: S4 Fig — (DOCX) [file ppat.1014424.s004.docx]

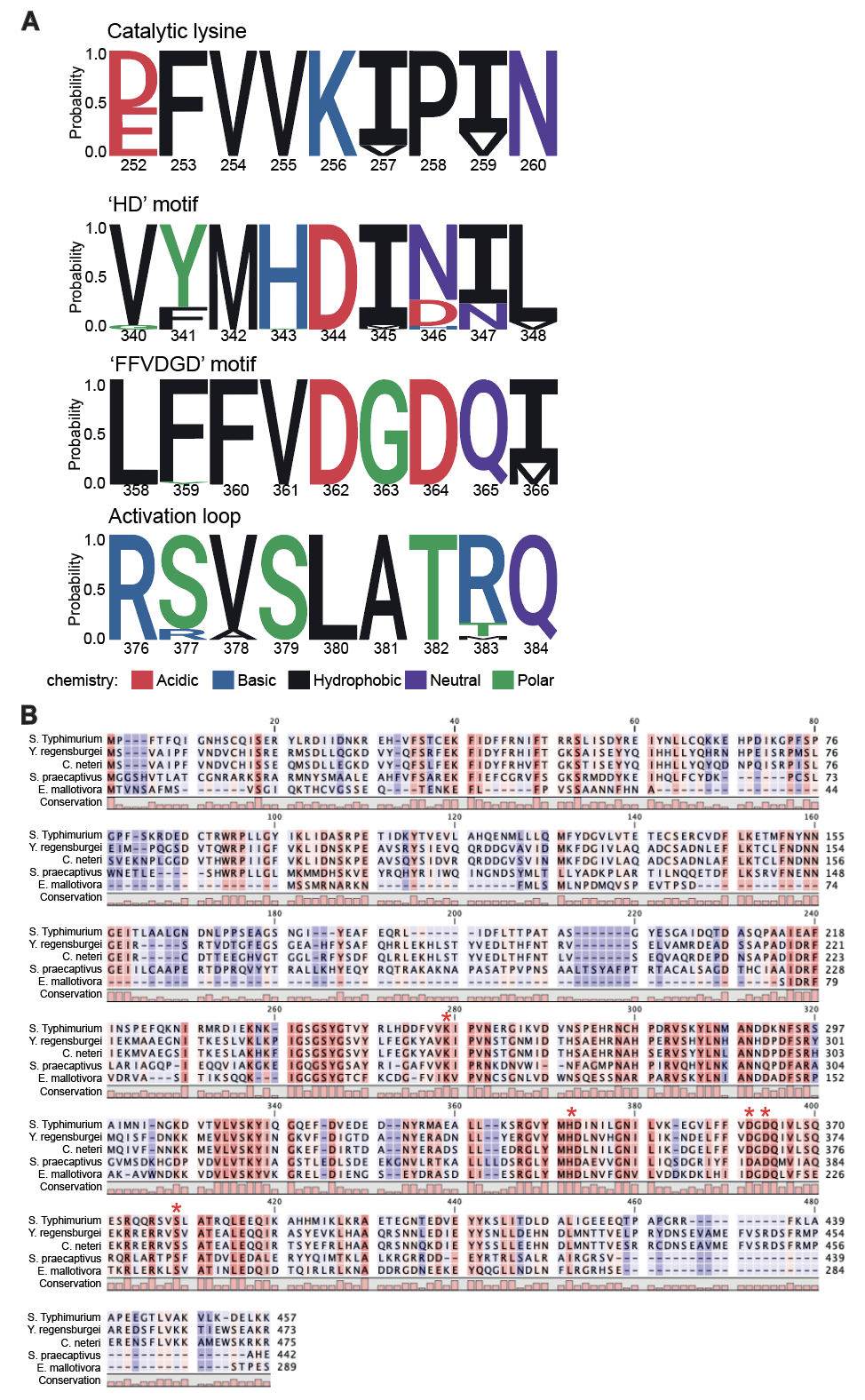


#### **S4 Fig: Sequence conservation across putative homologs of SteC**

1. Regions of interest were compared in the same 879 genomes from *Salmonella* clinical isolates analysed in **Fig 4A**. Sequence logos for these motifs were generated using the ggseqlogo package in R^35^.
2. A multiple sequence alignment was performed of the amino acid sequences of SteC from *Salmonella* Typhimurium strain 14028s and its top four bacterial homologs identified by BLASTp search: *Yokenella regensburgei* (98% coverage, 41% amino acid identity), *Cedecea neteri* (92%, 42%), *Sodalis praecaptivus* (86%, 34%) and *Erwinia mallotivora* (42%, 49%) using T-Coffee in CLC Sequence Viewer 7. The background colour scale represents a spectrum from full homology (red) to complete lack of homology (light blue). The conservation graph below demonstrates the number of amino acid sequences sharing any given residue. The catalytically essential residues in SteC, K256, H343, D344, D362, D364 and S379, are annotated with a red asterix, demonstrating conservation across the homologs.
