# Supplementary material for: Salmonella effector kinase SteC is activated by phosphorylation at Serine 379
Source: PLoS Pathog. 2026 Jul 16;22(7):e1014424. doi: 10.1371/journal.ppat.1014424 (PMC13395416; doi:10.1371/journal.ppat.1014424)
Supplement: S1 Table — (DOCX) [file ppat.1014424.s005.docx]

#### **S1 Table: Phosphorylated peptides of FMNL1 after incubation with SteC and ATP**

FMNL1_1-458_ and SteC_1-457_ were expressed in Sf9 cells. FMNL1 was analysed by phospho-MS either alone or after incubation with SteC and ATP. No FMNL1 phosphosites were detected in the control condition. Phosphorylated FMNL1 peptides after incubation with SteC and ATP are reported here. Data were analysed with MaxQuant^28^.

| **Starting amino acid** | **Peptide sequence** | **Mass (Da)** | **Phosphorylation site** |
| --- | --- | --- | --- |
| 178 | NKPLEQ**pS**VEDLSK | 1565.73 | S185 |
| 178 | NKPLEQ**pS**VEDLSKGPPSSVPK | 2315.14 | S185 |
| 191 | GPPSSVPK**pS**R | 1090.56 | S199 |
| 199 | SRHL**pT**IK | 933.48 | T203 |
| 199 | SRHL**pT**IKLTPAHSR | 1695.89 | T203 |
| 201 | HL**pT**IKLTPAHSRK | 1580.86 | T203 |
| 201 | HL**pT**IKL**pT**PAKSR | 1532.73 | T203 & T207 |
