# Supplementary material for: Salmonella effector kinase SteC is activated by phosphorylation at Serine 379
Source: PLoS Pathog. 2026 Jul 16;22(7):e1014424. doi: 10.1371/journal.ppat.1014424 (PMC13395416; doi:10.1371/journal.ppat.1014424)
Supplement: S2 Table — (DOCX) [file ppat.1014424.s006.docx]

**S2 Table: S379 phosphorylation in SteC by expression method, amino acid range and incubation with ATP**

SteC was expressed in *E. coli* or Sf9 cells and was analysed either alone or after incubation with ATP (5mM) for 30 minutes. The peptide spanning S377 to R383 was identified in phosphorylated and unphosphorylated forms. The phosphorylated form was found to be phosphorylated at S379 (**Figure 2A**). Peptide intensity is reported as multiples of 10^6^ intensity units. Data were analysed with Skyline. NaN, no peptide detected.

| **Protein** | **Expression system** | **Incubation with ATP** | **Intensity of peptide** | | **Proportion phosphorylated**  (% total) |
| --- | --- | --- | --- | --- | --- |
|  |  |  | SVSLATR | SV**pS**LATR |  |
| **SteC_210-457_** | *E. coli* | No | 42824 | 32702 | 43.3 |
|  |  | Yes | 42818 | 37021 | 46.4 |
| **SteC_210-457_ K256H** | *E. coli* | No | 41455 | NaN | 0 |
|  |  | Yes | 232.49 | NaN | 0 |
| **SteC_210-429_** | E. coli | No | 74966 | 28460 | 27.5 |
|  |  | Yes | 71175 | 22047 | 23.6 |
| **SteC_1-457_** | Sf9 | No | 143.14 | 11782 | 98.8 |
|  |  | Yes | 71.900 | 10820 | 99.3 |
| **SteC_1-457_ K256H** | Sf9 | No | 84179 | 58413 | 87.4 |
|  |  | Yes | 86524 | 65901 | 88.4 |
