# Supplementary material for: Salmonella effector kinase SteC is activated by phosphorylation at Serine 379
Source: PLoS Pathog. 2026 Jul 16;22(7):e1014424. doi: 10.1371/journal.ppat.1014424 (PMC13395416; doi:10.1371/journal.ppat.1014424)
Supplement: S3 Table — (DOCX) [file ppat.1014424.s007.docx]

**S3 Table: Phosphorylated and unphosphorylated peptides of recombinant SteC with and without incubation with ATP**

SteC_1-457_ expressed in Sf9 cells was analysed either alone or after incubation with ATP (5 mM). The peptide intensity for three phosphorylation peptides, with the corresponding non-phosphorylated peptides, with and without ATP, is reported in a logarithmic scale. Data were analysed with MaxQuant. NaN, no peptide detected.

| **Starting amino acid** | **Peptide sequence** | **Phosphorylation site** | **Intensity (-ATP)** | **Intensity**  **(+ATP)** |
| --- | --- | --- | --- | --- |
| 66 | EHPDIKGPFSPGPFSK |  | 6.52263 | 7.67358 |
| 66 | EHPDIKGPF**pS**PGPFSK | S75 | NaN | 7.25220 |
| 72 | GPFSPGPFSK |  | 8.92893 | 8.82299 |
| 72 | GPF**pS**PGPFSK | S75 | 7.20550 | 8.09496 |
| 377 | SVSLATR |  | 7.60412 | 7.28526 |
| 377 | SV**pS**LATR | S379 | 9.35727 | 9.25940 |
