# Supplementary material for: Salmonella effector kinase SteC is activated by phosphorylation at Serine 379
Source: PLoS Pathog. 2026 Jul 16;22(7):e1014424. doi: 10.1371/journal.ppat.1014424 (PMC13395416; doi:10.1371/journal.ppat.1014424)
Supplement: S1 File — (PDF) [file ppat.1014424.s008.pdf]

Figure 1b

Coomassie

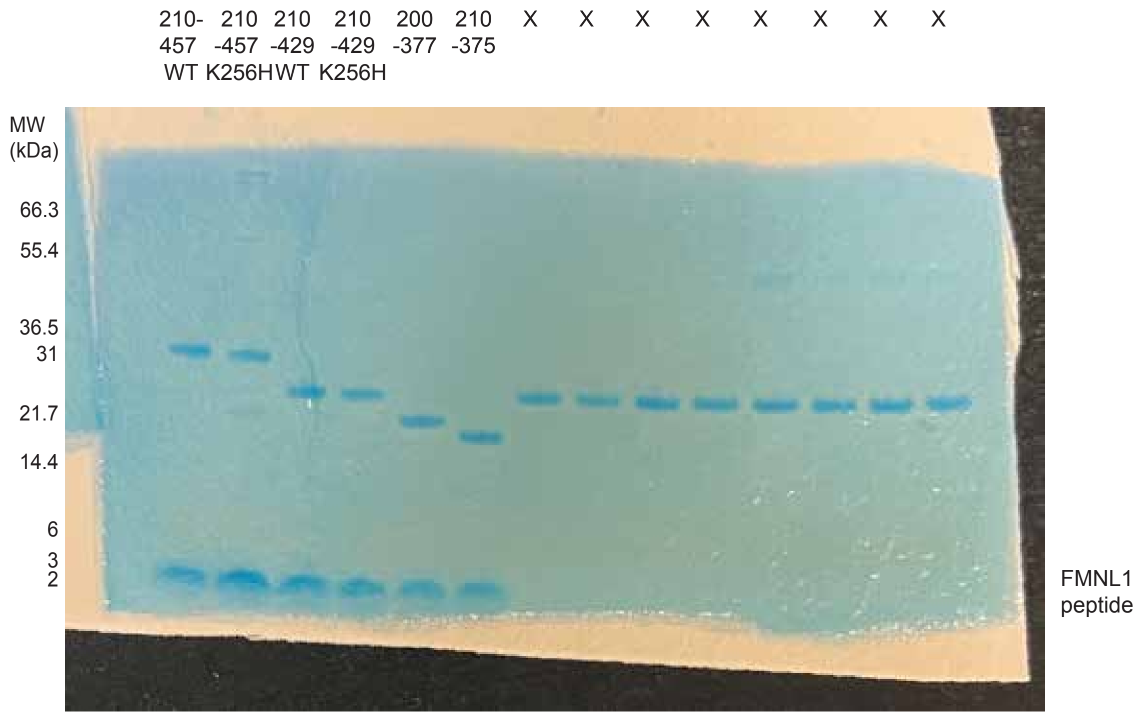

Autoradiography

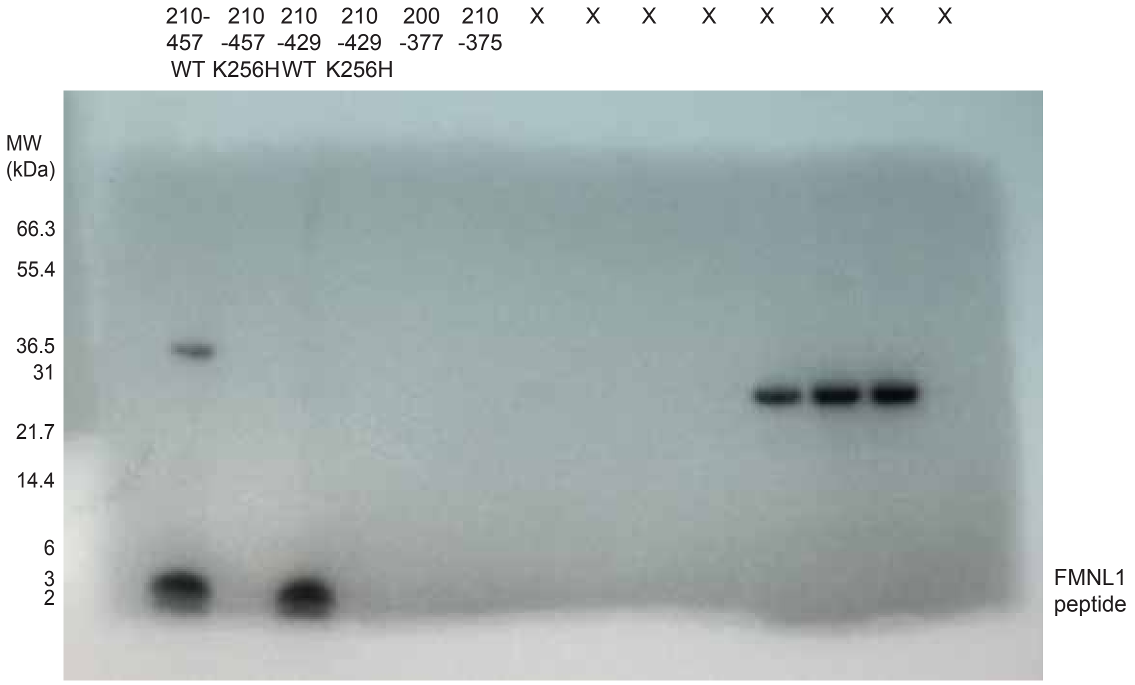

Figure 2c

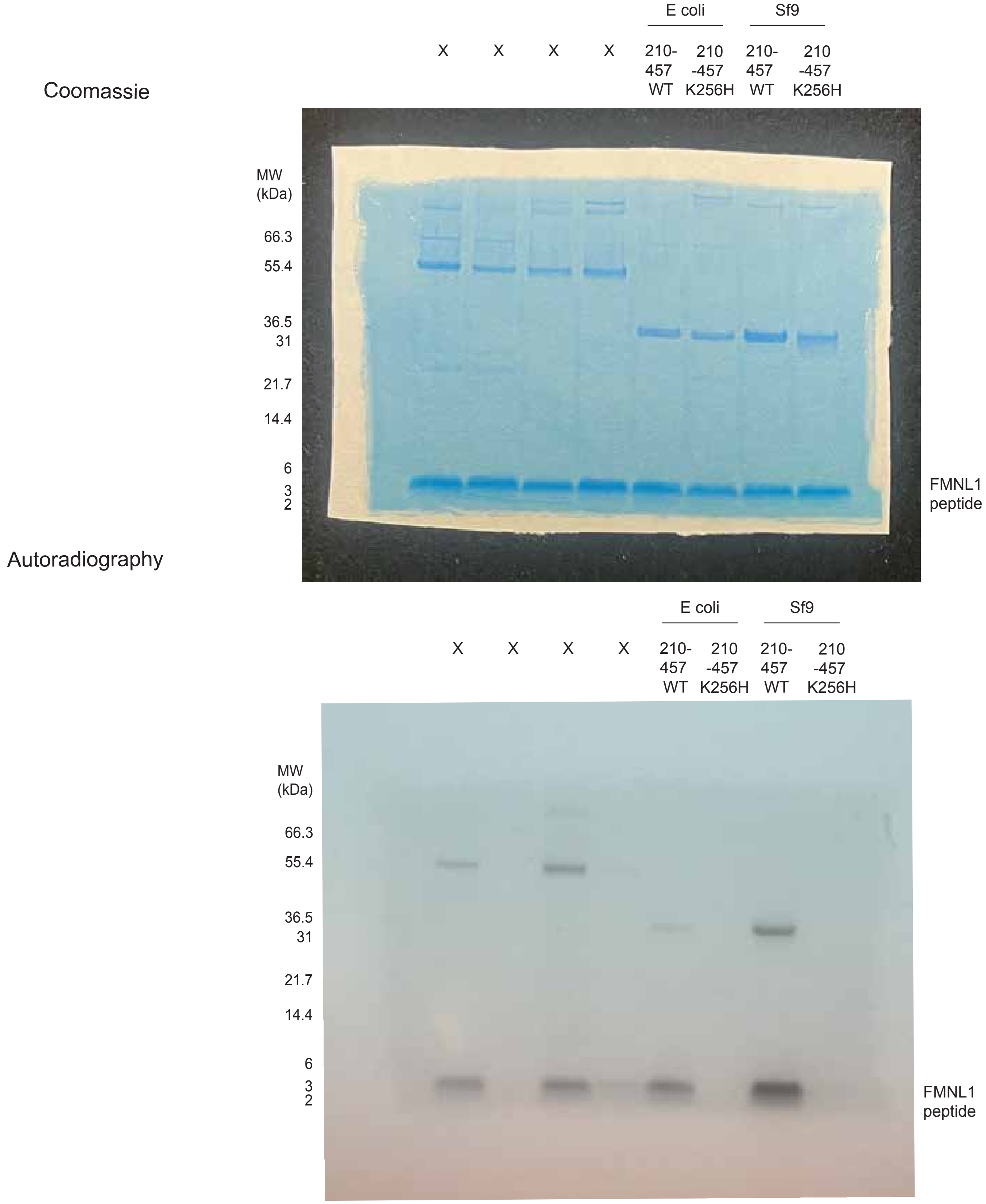
Coomassie
E coli
Sf9
X X X X
210-457 WT
210 -457 K256H
210-457 WT
210 -457 K256H
MW (kDa)
66.3
55.4
36.5
31
21.7
14.4
6
3
2
FMNL1 peptide
Autoradiography
E coli
Sf9
X X X X
210-457 WT
210 -457 K256H
210-457 WT
210 -457 K256H
MW (kDa)
66.3
55.4
36.5
31
21.7
14.4
6
3
2
FMNL1 peptide

Figure 2e

Coomassie

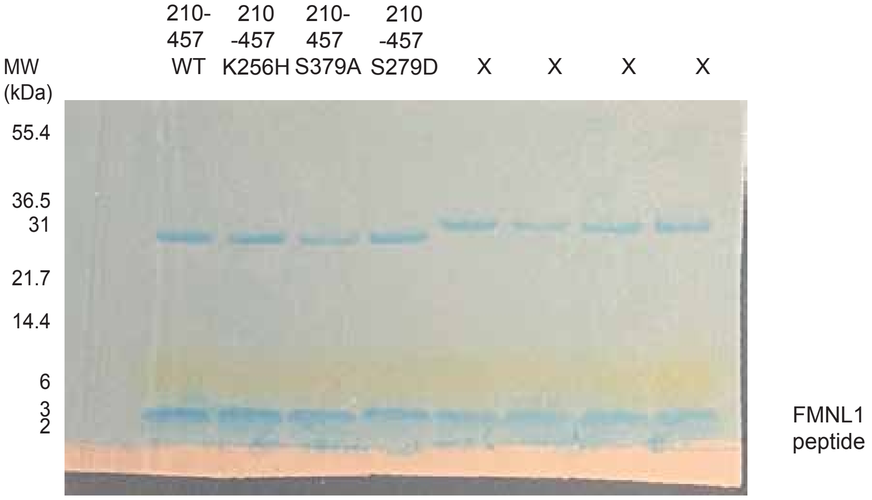

Autoradiography

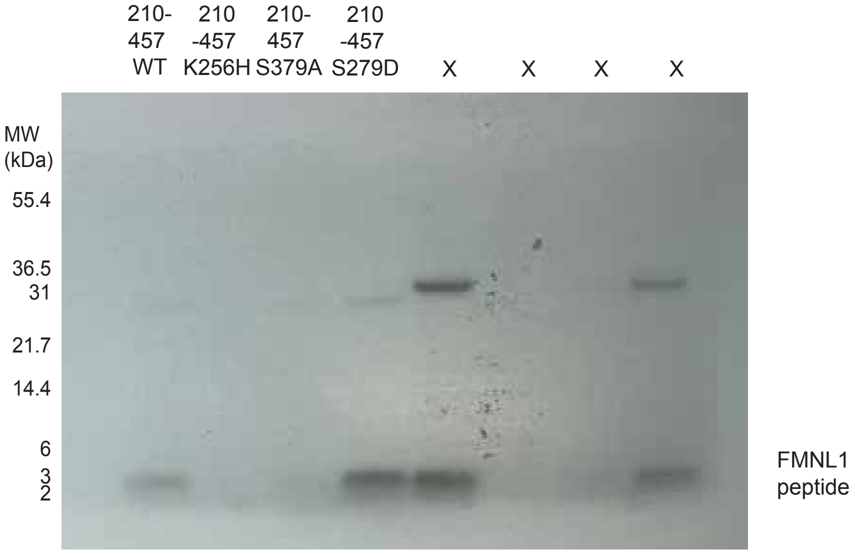

Figure 2f

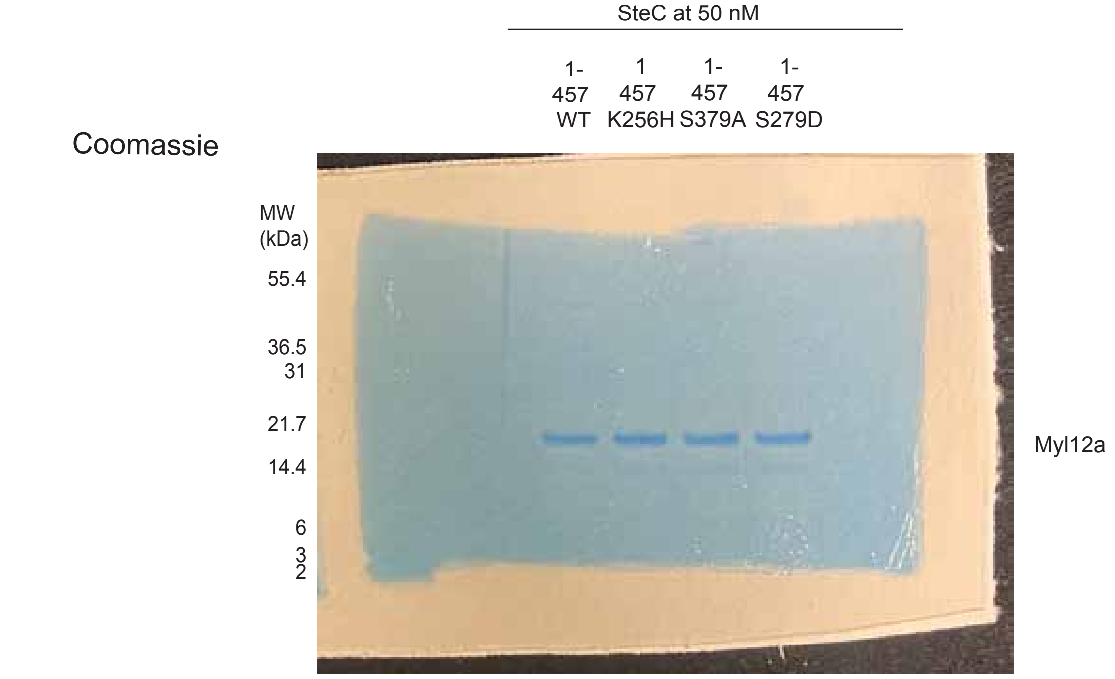
SteC at 50 nM
1-457 WT
1 457 K256H
1-457 S379A
1-457 S279D
Coomassie
MW (kDa)
55.4
36.5
31
21.7
14.4
6
3
2
Myl12a

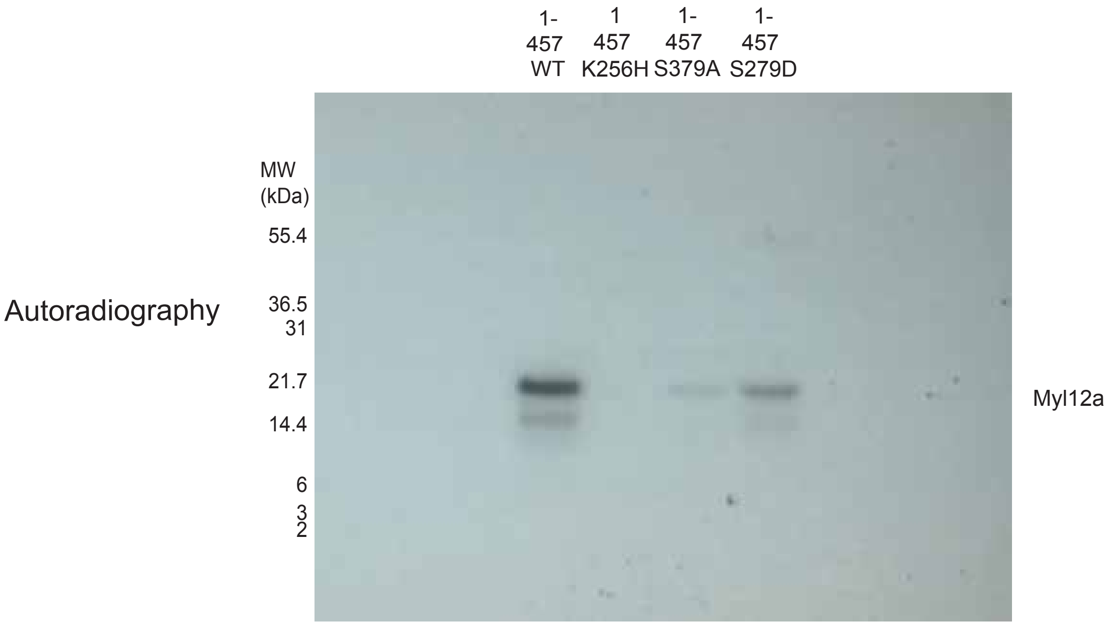
1-457 WT
1 457 K256H
1-457 S379A
1-457 S279D
Autoradiography
MW (kDa)
55.4
36.5
31
21.7
14.4
6
3
2
Myl12a

Figure 2h

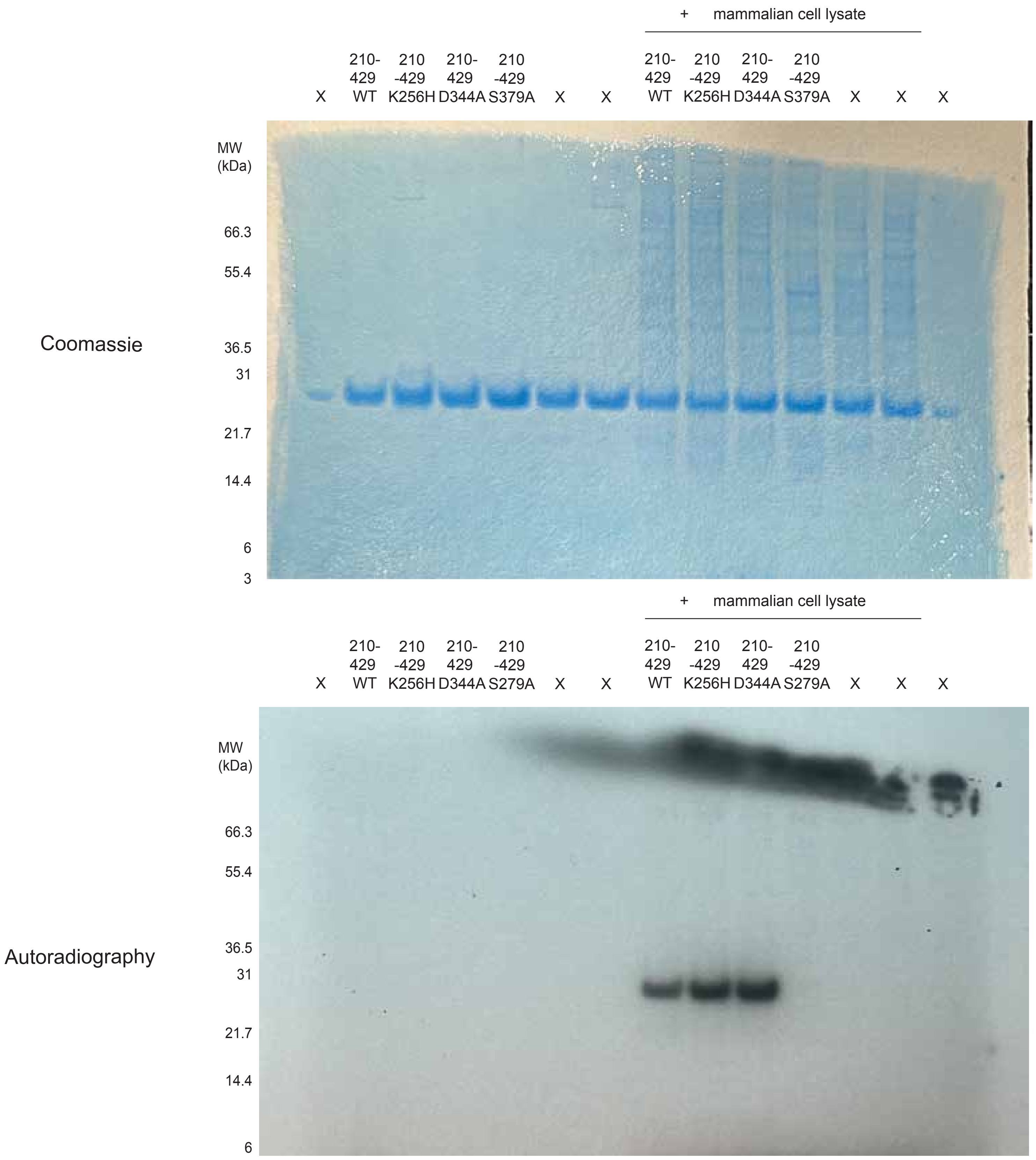

+ mammalian cell lysate
X
210-429 WT
210-429 K256H
210-429 D344A
210-429 S379A
X
X
210-429 WT
210-429 K256H
210-429 D344A
210-429 S379A
X
X
X
Coomassie
MW (kDa)
66.3
55.4
36.5
31
21.7
14.4
6
3
+ mammalian cell lysate
X
210-429 WT
210-429 K256H
210-429 D344A
210-429 S279A
X
X
210-429 WT
210-429 K256H
210-429 D344A
210-429 S279A
X
X
X
Autoradiography
MW (kDa)
66.3
55.4
36.5
31
21.7
14.4
6

Figure 3c

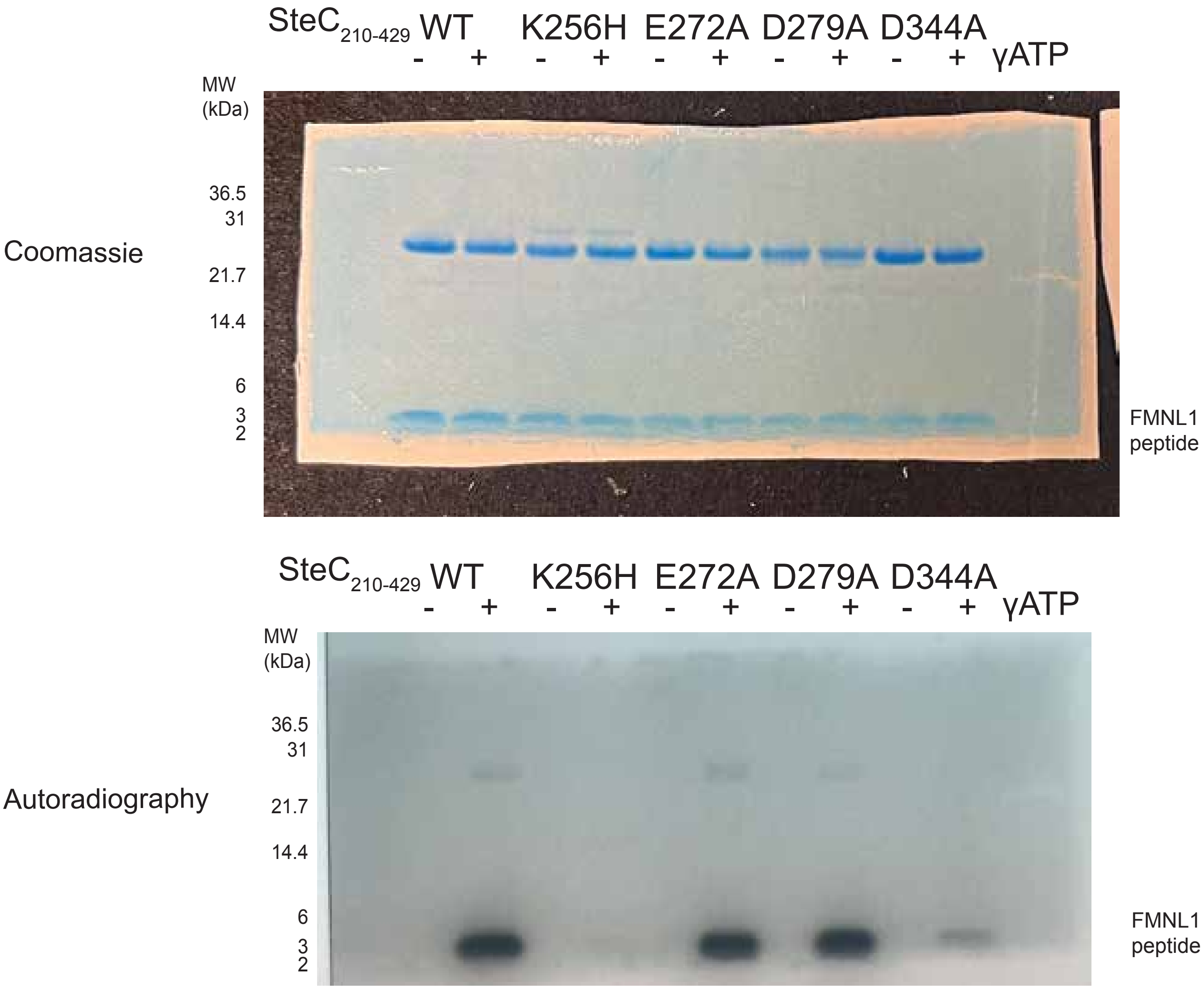
SteC$_{210-429}$ WT K256H E272A D279A D344A
- + - + - + - + - + γATP
MW (kDa)
36.5
31
21.7
14.4
6
3
2
Coomassie
FMNL1 peptide
SteC$_{210-429}$ WT K256H E272A D279A D344A
- + - + - + - + - + γATP
MW (kDa)
36.5
31
21.7
14.4
6
3
2
Autoradiography
FMNL1 peptide

Figure S1c and S2a

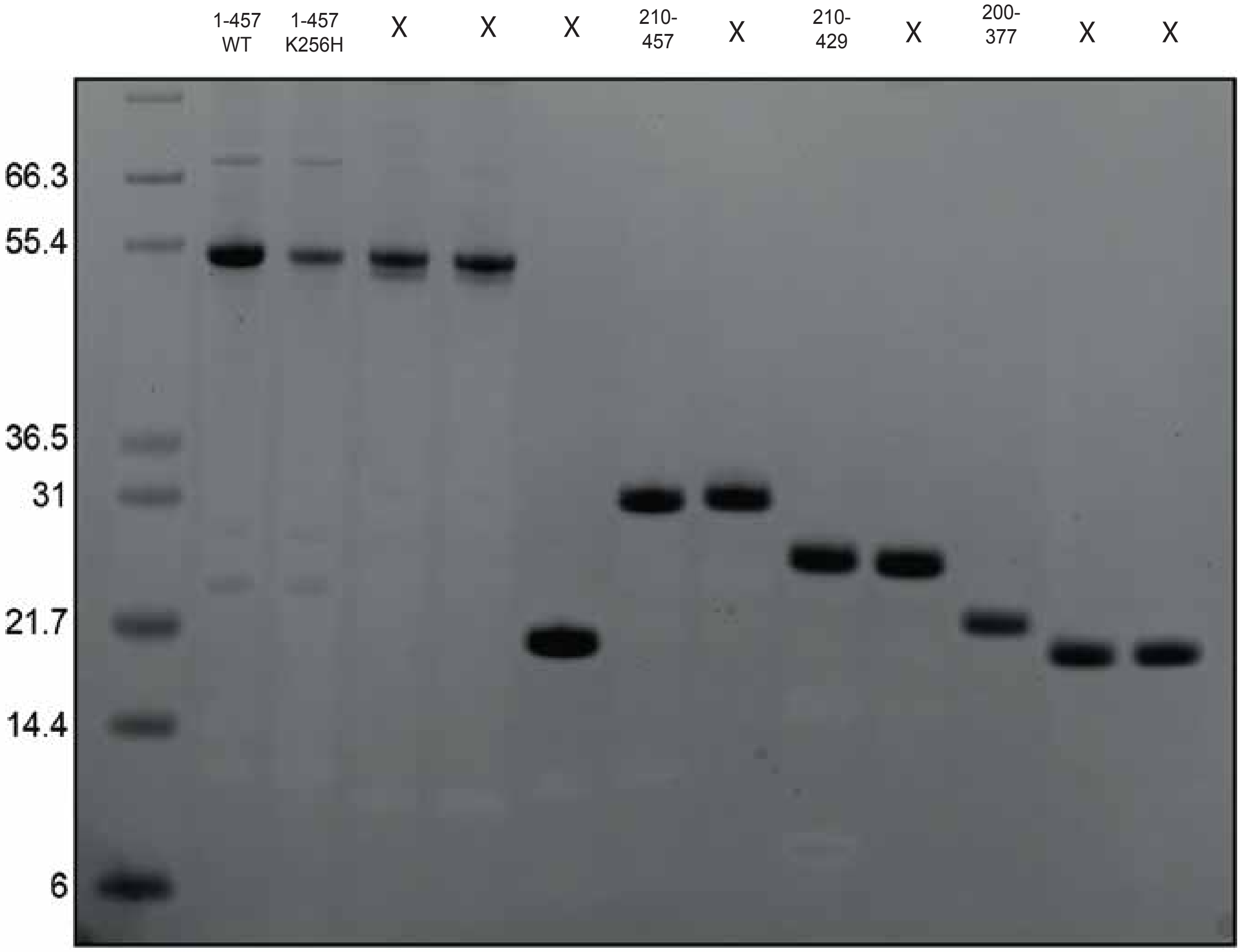

Coomassie

Figure S3d

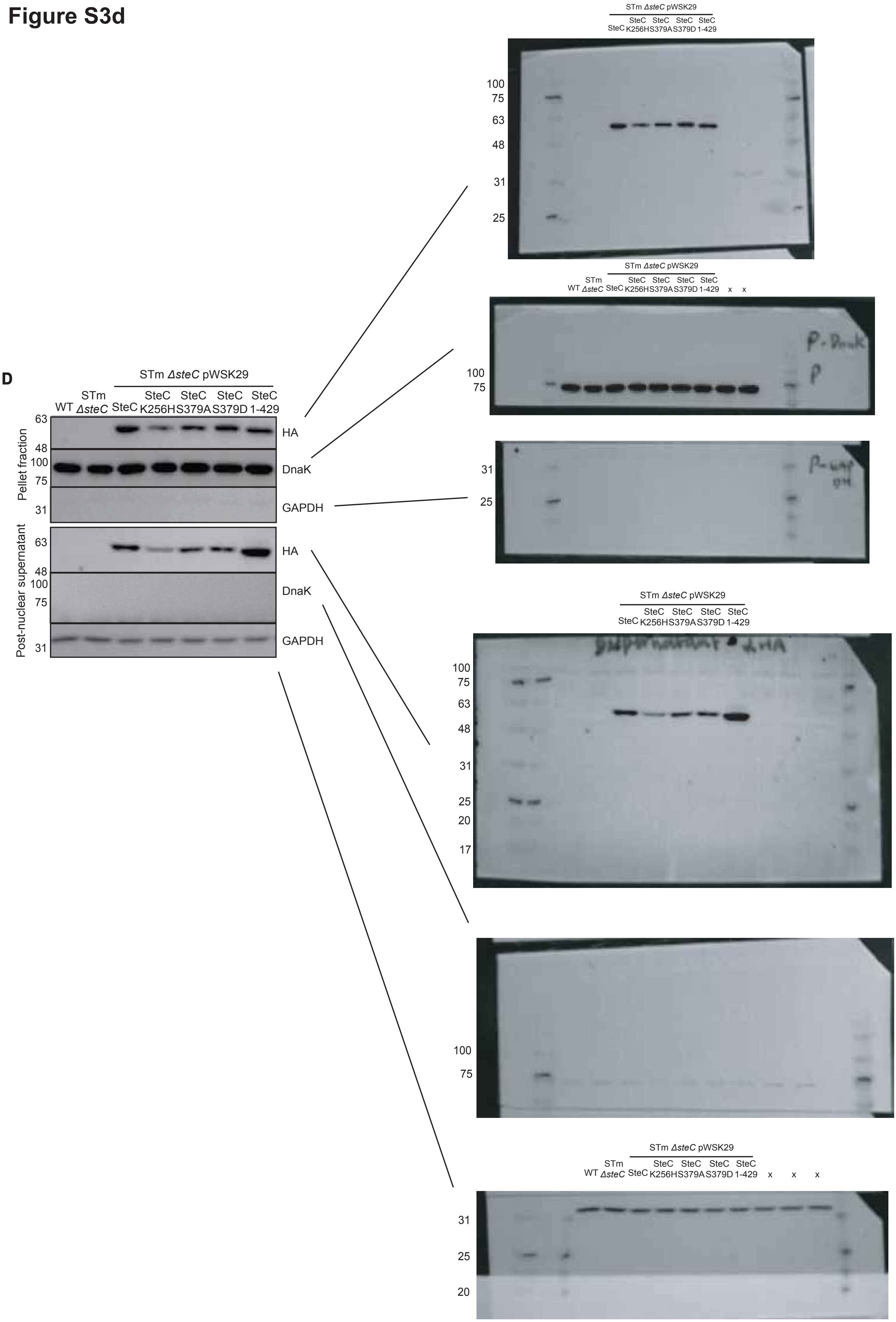

D
STm ΔsteC pWSK29
WT
STm ΔsteC
SteC
SteC K256H
SteC S379A
SteC S379D
SteC 1-429
Pellet fraction
Post-nuclear supernatant
HA
DnaK
GAPDH
63
48
100
75
31
25
20
17
x

Figure S1c
