# Supplementary material for: Salmonella effector kinase SteC is activated by phosphorylation at Serine 379
Source: PLoS Pathog. 2026 Jul 16;22(7):e1014424. doi: 10.1371/journal.ppat.1014424 (PMC13395416; doi:10.1371/journal.ppat.1014424)
Supplement: Attachment — Submitted filename: Response to reviewers.pdf [file ppat.1014424.s009.pdf]

Point by point response for manuscript: PPATHOGENS-D-26-00083

We thank the reviewers for their supportive and insightful comments on our manuscript. We have responded to all the issues raised as detailed in our point-by-point response below. Specifically, we have tried to be more explicit and clear about the two different conditions under which we assessed phosphorylation of SteC: 1) phosphorylation that occurs during protein expression, either by a eukaryotic kinase or through autophosphorylation that happens in a co-translational manner, versus 2) phosphorylation taking place in kinase assays in the presence of radioactive ATP, which was used to monitor substrate phosphorylation by purified proteins.

Response to reviewers' comments

Reviewer #1: Assuming the affinity of mant-AMPPNP equals the affinity of ATP, a difference of ~2-fold (5.4 and 11.8 μM) cannot be of biological/biochemical significance. Concentrations of ATP in cells are around 1 millimolar, and such tiny difference in affinity won't produce any meaningful change in "activation". The authors must remove that conclusion from the paper, specifically page 11 lines 247-249. Their data suggests the opposite of what the authors concluded, that the S379D mutation in fact has only minimal effect on SteC activity.

We thank the reviewer for highlighting this. We now realise that our description of these experiments and explanation wasn't sufficiently clear. What we wanted to say is that WT $SteC_{210-429}$, which becomes partially phosphorylated at S379 when expressed in *E. coli* (Figure 2B) and the mutant phosphomimetic form, $SteC_{210-429;S379D}$ have very similar affinities (5.4 and 11.8 μM). In contrast, $SteC_{210-429;S379A}$, that cannot be phosphorylated at S379 has a much lower affinity for the nucleotide, which is too low to fit to a curve. Therefore, we propose that the phosphorylation of S379 activates SteC by increasing its affinity for ATP.

We have amended lines 256-265, which we hope has clarified what we are trying to say.

Reviewer #2: -There is inconsistency in assay conditions, which may have been contributed to some confusion on my end in interpreting the results. But in general different SteC constructs are used (full length, 210–457, 210–429, 200–377) and it isn't clearly explained why.

We appreciate that we had not properly explained the reason for using these different constructs. We have now provided additional rationale to construct design in lines 129-134, highlighting why we designed the different constructs. To summarise here:

210-457 represents the complete kinase domain

210-429 lacks the predicted C-tail, which was of unknown function

200-377 and 210-375 lack αF and αG as well as the C-tail and resemble the recently crystallised form of SteC.

Furthermore, we now provide additional descriptions of these proteins in the methods section (lines 601-604), highlighting that $SteC_{210\text{-}457}$ was more aggregation-prone than $SteC_{210\text{-}429}$. Therefore, when larger amounts of protein were required, for example for the experiments shown in Figure 2H, we analysed $SteC_{210\text{-}429}$. A direct comparison of $SteC_{210\text{-}457}$ and $SteC_{210\text{-}429}$ was conducted and is shown in Figure 1B, which demonstrated that *in vitro*, both variants induced a comparable degree of FMNL1 peptide phosphorylation.

It isn't always clear which purification system has been used (E. coli versus insect cells).

The reviewer is correct and we apologise for not being explicit. *E. coli* expression was used unless otherwise stated. To provide greater clarity, we have now added the expression system to all Figure legends and to the relevant text.

In addition, kinase assay conditions differ (e.g., substrate concentrations of 5 µM versus 100 µM;

The protein substrate concentration used was 5 µM as standard when investigating phosphorylation of protein substrates. However, for the low molecular weight peptide substrate, 100 µM was used to enable visualisation by Coomassie staining. We have now explained this in the revised manuscript (lines 679-686).

ATP inclusion or omission are not always clearly explained

We thank the reviewer for offering us the opportunity to provide greater clarity. In the revised manuscript we have adjusted the wording in the main text to avoid ambiguity (e.g. line 179) and included details regarding the addition of ATP in the appropriate figure legends.

As previously demonstrated, and also shown here (Fig. 1B), SteC undergoes autophosphorylation when purified from E. coli. It is therefore unclear how the authors definitively distinguish autophosphorylation from host-kinase-mediated phosphorylation. What is the identity of the autophosphorylation site observed in Fig. 1B? Is this site S379? If SteC purified from E. coli is capable of autophosphorylation and activity, how important is host kinase phosphorylation?

This reviewer is correct in pointing out that some level of autophosphorylation at S379 occurs during expression in *E. coli* (see Figure 2B). However, we believe that this is a co-translational event and an activity that is largely lost once the protein is fully folded (see Figure 2B, hardly any change +/- ATP). Such co-translational autophosphorylation events have been reported for other kinases (see reference

18 and a new reference 19 which has been added to support this claim). No autophosphorylation is observed in the K256H kinase deficient mutant upon expression in *E. coli*. In contrast, upon expression in insect cells we see a high level of S379 phosphorylation even in the presence of the K256H mutant, which must hence be from a host kinase. The importance of S379 phosphorylation for substrate phosphorylation is highlighted in Figure 2E that clearly shows that the S379D mutant protein is much more active than the S379A mutant and even the WT protein.

Concerning the low level of autophosphorylation detected in the radioactive kinase assay shown in Figure 1B, we believe that this is a very low level of autophosphorylation, potentially at a non-specific site, that may not have been covered in our phospho-mapping experiments.

In Fig. 2B, the quantification of S379 phosphorylation across expression systems is difficult to interpret. It is not clear that all primary data are shown, and the explanation in the text is somewhat confusing.

To address this comment, we have added **new Supplementary Table 2** to the revised manuscript, showing the intensity of peptide signal for non-phosphorylated and phosphorylated S379 containing peptides across the different conditions reported in Figure 2B. We have also revised our explanation in the text, introducing first the data from *E. coli* purified SteC constructs and then introducing the insect purified constructs.

2C shows that SteC phosphorylation of its substrate is increased when SteC is purified from insect cells, but this experiment doesn't include the SteC phosphorylation by autoradiography, so it is difficult to see how much insect cell purification contributes to SteC phosphorylation.

The data shown in Figure 2B indicate that SteC is already fully phosphorylated on S379 when expressed in insect cells and addition of ATP to the purified protein doesn't further enhance the signal. Together with the observation that the kinase-deficient SteC mutant K256H also shows almost complete phosphorylation on S379 upon expression in insect cells (which is not further enhance upon addition of ATP), this suggests that S379 is phosphorylated by a eukaryotic kinase that is present in insect cells. For this reason, we did not include an autoradiogram of SteC phosphorylation as the data shown in Figure 2B indicate that this does not occur due to complete phospho-modification of S379 during insect cell expression.

Here (2C) the S379 mutation is not tested. S379 is tested in 2E but this is confusing because in that experiment SteC is purified from E. coli, therefore showing that S379 phosphorylation is not in this context mediated by a host kinase? Why is SteC not autophosphorylated in these conditions (when it was in 1B)?

This is a very good point, and we think the most likely explanation is that *E. coli* expressed SteC has very low autophosphorylation activity that is not always detected in radioactive kinase assays, depending on the exposure time during blot development, which is influenced by the strongest signal on the blot (generally the FMNL1 substrate). For this reason, it is not possible to compare absolute signal strength across different experiments, only within the same blot.

To illustrate this point we have carried out an autophosphorylation assay with *E. coli* expressed SteC 210-457 WT and kinase-deficient K256H, which allows long exposure times to visualise small amounts of autophosphorylation of the WT protein.

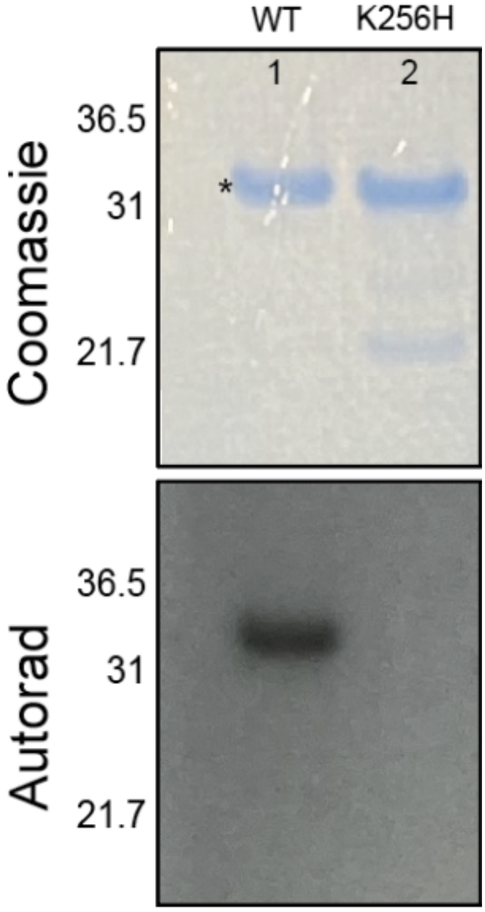

Relating this back to 2E, the main observation is that the phosphomimetic variant of SteC (S379D), which is more active than the WT protein, also undergoes some auto-phosphorylation, which we speculate is at a non-specific site we have not detected in our mass spectrometry experiments.

Fig. 2H does suggests that murine cell lysate can phosphorylate SteC K256H, consistent with host-mediated phosphorylation. However, I find the experiment confusing and somewhat inconsistent with the rest of the figure: Why is FMNL1 included in the control condition? Why is a different SteC construct (210–429) used

here compared to other assays? Why does SteC not appear to autophosphorylate in this assay (left)?

We thank the reviewer for highlighting this error in the figure legend. There is no FMNL1 in this assay and the figure legend has been corrected accordingly.

$SteC_{210-429}$ was used in this experiment as whilst it retains activity towards substrates there is either very low (Figure 3C) or no autophosphorylation detected (Figure 1B, 2H), providing a greater dynamic range to detect phosphorylation caused by the addition of the mammalian cell lysate.

Most importantly, if SteC activation is indeed mediated by a host kinase, this kinase should be identified or at least narrowed down. At present, no kinase inhibitors or genetic knockouts are tested, no consensus motif analysis is presented, no candidate kinases are proposed, no infection-based phospho-analysis is shown. Given that host-mediated activation is the central conceptual advance of the manuscript, the lack of identification of the responsible kinase (and how it affects Salmonella infection/actin polymerisation) substantially limits impact.

We agree that identifying the host kinase would significantly extend the impact of our findings. However, the identification of an upstream kinase is a difficult and laborious process especially in the absence of candidate kinases for which specific inhibitors are available. Therefore, we believe this would go beyond the scope of this study.

Nevertheless, we did perform motif analysis, and the findings of this are shown below:
Using the Kinase Library site (https://kinase-library.phosphosite.org/kinase-library/score-site) we analysed the S379 phosphosite of SteC with 7 amino acids either side (SRQQRSVpSLATRQLE). This suggested 49 human kinases with an >90% score that might be able to phosphorylate this site. See the screenshot below. Given this large number of potential upstream kinases, we don't think inclusion of these data would add to the manuscript.

Kinase	Gene Name	Kinase Family	Log_2(Score)	Score Rank	Site Percentile	Percentile Rank
PHKG2	PHKG2	CAMK	2.8884	3	98.66	1
TTBK1	TTBK1	CK1	1.2802	34	98.06	2
TTBK2	TTBK2	CK1	1.4313	26	97.85	3
PAK6	PAK6	STE	3.2599	2	97.55	4
CHK2	CHEK2	CAMK	3.2949	1	96.58	5
IKKA	CHUK	Other	1.3746	27	96.45	6
PHKG1	PHKG1	CAMK	1.8097	18	96.42	7
PAK4	PAK4	STE	2.2598	9	96.24	8
PAK5	PAK5	STE	2.3191	7	96.1	9
PKN1	PKN1	AGC	1.9631	15	95.71	10
PKN2	PKN2	AGC	1.8758	17	95.42	11
IRAK1	IRAK1	TKL	0.4322	75	95.39	12
MAPKAPK5	MAPKAPK5	CAMK	2.0466	11	95.17	13
S6K1	RPS6KB1	AGC	2.5167	5	95.04	14
SRPK3	SRPK3	CMGC	2.8322	4	94	15

Filtering by Percentile Rank (asc) 19 Max 50

Kinome Tree

DOWNLOAD

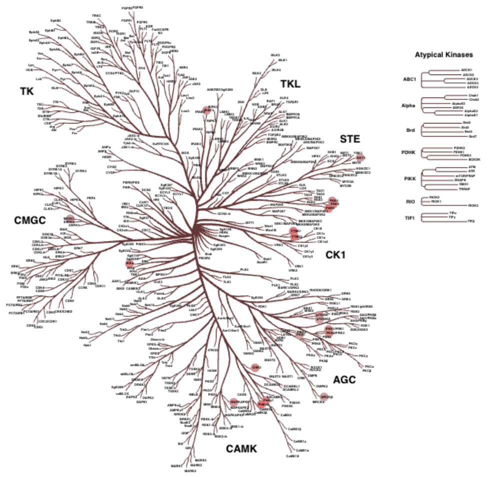

Finally, the discussion of the C-terminal tail is unclear and seems disconnected from the rest of the study. The discrepancy between in vitro activity and cellular phenotype is intriguing but insufficiently explained. The mechanistic role of the C-tail during infection remains unresolved and somewhat obscures the central narrative.

> This manuscript presents two novel findings that advance the field. The first is that SteC is phosphorylated at S379 by a eukaryotic kinase and phosphorylation at this residue is necessary for its full kinase activity. Second, we demonstrate that even though the SteC C-tail is dispensable for kinase activity *in vitro* and SteC induced actin polymerisation upon ectopic expression within eukaryotic cells, it is essential for activity when SteC is delivered in mammalian cells from *Salmonella*. Despite not being able to provide a mechanistic role for the C-tail, including these findings in our manuscript ensures that the community can build on this intriguing observation.

Part III – Minor Issues: Editorial and Data Presentation Modifications

Please use this section for editorial suggestions as well as relatively minor modifications of existing data that would enhance clarity.

Reviewer #1: Figures can be tightened up. For example, Fig. 1 D has the legend repeated three times and the units of protein concentration (mg/mL) repeated 18 times.

As this error is not in our uploaded source document, we suspect it arose from a formatting error. We will ensure that the newly uploaded documents are formatted correctly when converted to PDF.

"K" in "Kd" must be italicized.

This has been corrected.

Reviewer #2: (No Response)
