# Supplementary material for: Salmonella effector kinase SteC is activated by phosphorylation at Serine 379
Source: PLoS Pathog. 2026 Jul 16;22(7):e1014424. doi: 10.1371/journal.ppat.1014424 (PMC13395416; doi:10.1371/journal.ppat.1014424)
Supplement: Attachment — Submitted filename: Response_to_Reviewers_Round2.pdf [file ppat.1014424.s010.pdf]

Additional Editor Comments:

One reviewer highlights that some of their comments remain unanswered, and the paper's conclusions and title have not been edited accordingly. I would like to give you another opportunity to thoroughly address their comments. If you deemed it impossible to answer, your best next move would be to start again elsewhere at this point, as it is unlikely that the reviewer will change their mind.

We'd like to thank the editor for giving us the opportunity to respond to the remaining concerns of reviewer 2 and to make further edits to the manuscript. These are freshly tracked on a version that is marked in yellow to demonstrate the first round of edits alongside, and include changes to the title and conclusion.

Reviewers' Comments:

Reviewer's Responses to Questions

Part I - Summary
Please use this section to discuss strengths/weaknesses of study, novelty/significance, general execution and scholarship.

Reviewer #1: The authors have carefully and successfully addressed my concerns.

Reviewer #2: I appreciate the authors' efforts to clarify several of the points raised in the initial review, and the revised manuscript is clearer in parts. However, while some of the inconsistencies are discussed, they are not fully resolved. Moreover, the central conclusion and title of the study are that SteC is phosphorylated by a host kinase. While the data support host-mediated phosphorylation, they also indicate that autophosphorylation at S379 occurs at levels that are at least partially comparable, and this is not fully reconciled with the overall interpretation.

We had believed that the edits made in our first response to Reviewer 2 addressed this issue, specifically lines 183-203, 206-212 and 214-218. However, we have now made further edits, especially to the title, the abstract and conclusion (lines 411-421) to better reflect what the data show and our interpretation of the data.

To summarise our reasoning: Firstly, auto-phosphorylation can occur, as evidenced by phosphorylated S379 being detected in bacterially expressed protein in a kinase-activity dependent manner (Figure 2B). Secondly, this must occur co-translationally during protein folding, because the relative amount of pS379 does not increase when the purified protein is incubated with ATP (Figure 2B). Finally, phosphorylation at S379 by a eukaryotic kinase must also occur and is more efficient based on mass spectrometry of insect cell expressed protein, with evident phosphorylation occurring even on the kinase inactive mutant.

Part II – Major Issues: Key Experiments Required for Acceptance
Please use this section to detail the key new experiments or modifications of existing experiments that should be absolutely required to validate study conclusions.

Generally, there should be no more than 3 such required experiments or major modifications for a "Major Revision" recommendation. If more than 3 experiments are necessary to validate the study conclusions, then you are encouraged to recommend "Reject".

Reviewer #1: N/A

Reviewer #2: 1-Autophosphorylation versus host-mediated phosphorylation remains unclear
The data show substantial phosphorylation of S379 in SteC purified from E. coli (Fig. 2B). Even if these levels are 50% lower than in insect cells, they are dependent on the kinase activity of SteC. This is not sufficiently acknowledged in the interpretation.

We agree and accordingly have changed the title and the conclusion, as summarised above.

While the authors propose that this reflects co-translational autophosphorylation that is largely lost once the protein is folded, this explanation is not experimentally demonstrated and remains difficult to reconcile with the in vitro kinase assays.

Figure 2B tests the relative S379 phosphate occupancy of SteC expressed in *E. coli* before or after incubation with ATP. There is minimal increase in the amount of phospho-S379 detected after ATP incubation, indicating that fully folded SteC is not capable anymore to undergo autophosphorylation and hence that it must occur co-translationally. We had stated this in the previous revision (see lines 194-198).

We acknowledge that a low autophosphorylation signal is observed with *E.coli*-expressed protein upon incubation with ATP (see for example Figure 1B). This has already been commented on in the revised version, please see lines 198-202. We now also further comment on the relative contributions of co-translational autophosphorylation versus host kinase-induced phosphorylation in the summary, lines 411-421.

As a result, the conclusion that S379 phosphorylation is predominantly host-mediated remains insufficiently supported.

The conclusion that a host kinase can phosphorylate S379 comes from the same experiment, reported in Figure 2B. Insect cell expressed SteC is more heavily phosphorylated at S379 than *E. coli* expressed protein, and importantly this occurs on the kinase-inactive variant of SteC, unequivocally demonstrating that another kinase can phosphorylate SteC (see WT SteC versus kinase-dead).

We agree that we cannot state specifically how much of the completely phosphorylated SteC that can be purified upon expression in insect cells comes from co-translational autophosphorylation versus phosphorylation by a kinase from insect cells. Nevertheless, the observation that a kinase inactive mutant is nearly completely phosphorylated at S379 suggests that phosphorylation in trans by a kinase from insect cells is highly efficient. We have added additional text to the summary to be more explicit (please see lines 411-421).

Furthermore, Figure 2C compares insect- and *E. coli*-expressed $SteC_{210-457}$ in substrate phosphorylation assays. These data show clearly that *E. coli*-expressed protein (which is less phosphorylated at S379, Figure 2B) is also significantly less active than insect cell-expressed protein.

For example, Fig. 2E is used to demonstrate that S379 phosphorylation is essential for SteC activity against FMNL1, yet SteC is purified from E. coli in this context. This raises the question of how essential host kinase activity is for SteC function (This point was raised in the original review but was not addressed).

We believe that the essentiality of S379 phosphorylation to SteC function is clearly demonstrated with the combination of assays performed here *in vitro*, *in cellulo* and in infection studies following the reasoning explained above. However, we agree that we cannot conclusively determine the relative importance of co-translational autophosphorylation versus host kinase-mediated phosphorylation on S379. We would suggest that a fair interpretation of their relative importance comes from the analysis of phosphate occupancy at S379, comparing insect cell expressed full length WT SteC, or its kinase inactive variant, which is nearly completely phosphorylated at S379.

Given the importance of distinguishing host- versus auto-phosphorylation for the conclusions of this study, autoradiography for SteC phosphorylation should be consistently shown across all relevant experiments.

We feel that the more careful interpretation of the data in the latest revised version of manuscript, especially our changes to the title and conclusion (lines 411-421) mean that our interpretations are now fair and the data are not drawn out of context.

2- Inconsistency in constructs, systems, and assay conditions
While the authors have provided explanations for the use of different constructs, expression systems, and assay conditions, these inconsistencies remain. The explanations improve clarity but do not resolve the underlying issues.
For example, in Fig. 2B, the key comparison between SteC purified from E. coli and insect cells examines the proportion of phosphorylated protein, yet this comparison is not performed using the same SteC constructs. There may be valid reasons for this, but these are not clearly explained, making it difficult to interpret the comparison and

assess whether the observed differences reflect biological effects or differences in constructs.

We regret not having been able to compare identical constructs in Figure 2B. The reason is that full length SteC from *E. coli* could not be purified to the same standard as insect cell expressed protein. Even though the lower purity of full-length protein from bacteria did not interfere with the substrate phosphorylation assays shown in Figure 2F, the protein was not of sufficient quantity and purity for mass spectrometry analysis. We added a comment to the methods in lines 613-616 to explain this. Despite the forced asymmetry in this experiment, we feel the data is now accurately interpreted and there is no need for further experimental data to support the conclusions.

3- Host kinase claim remains insufficiently supported
While finding the specific upstream host kinase might be outside the scope of this study, the paper strongly emphasizes that host kinases activate SteC. Without identifying the kinase or doing functional validation (like testing inhibitor sensitivity, testing candidates), this part of the model isn't well supported.

We have amended the title to reduce the emphasis on a host kinase to instead focus on our finding that S379 is important for activity, which is supported by numerous experiments. However, when SteC is expressed from insect cells, compared to bacterial cells, we see more activity towards a substrate from the insect-expressed protein (Figure 2C) and Figure 2H shows that SteC can be phosphorylated by a host kinase. Therefore, our data suggest the presence of an upstream kinase and for these reasons we would like to propose this possibility within the manuscript and our amended discussion.

4- C-terminal tail section remains unclear
The discussion of the C-terminal tail remains insufficiently integrated with the rest of the study. While the discrepancy between in vitro activity and cellular phenotype is noted, the mechanistic role of the C-tail during infection remains unresolved and somewhat obscures the central narrative.

We respectfully disagree. We feel strongly that the data should be reported to the scientific community.

Part III – Minor Issues: Editorial and Data Presentation Modifications
Please use this section for editorial suggestions as well as relatively minor modifications of existing data that would enhance clarity.

Reviewer #1: N/A

Reviewer #2: (No Response)
