# Supplementary material for: Salmonella effector kinase SteC is activated by phosphorylation at Serine 379
Source: PLoS Pathog. 2026 Jul 16;22(7):e1014424. doi: 10.1371/journal.ppat.1014424 (PMC13395416; doi:10.1371/journal.ppat.1014424)
Supplement: Peer review history

# Peer review: Decision Letter 0

**DOI:** 10.1371/journal.ppat.1014424.r001

**Authors:** Sophie Helaine, Eva Heinz

23 Feb 2026

-->PPATHOGENS-D-26-00083

Salmonella effector kinase SteC is activated by host-mediated phosphorylation

PLOS Pathogens

Dear Dr. Rittinger,

Thank you for submitting your manuscript to PLOS Pathogens. After careful consideration, we feel that it has merit but does not fully meet PLOS Pathogens's publication criteria as it currently stands. Therefore, we invite you to submit a revised version of the manuscript that addresses the points raised during the review process.

Please submit your revised manuscript by Apr 24 2026 11:59PM. If you will need more time than this to complete your revisions, please reply to this message or contact the journal office at plospathogens@plos.org. When you're ready to submit your revision, log on to https://www.editorialmanager.com/ppathogens/ and select the 'Submissions Needing Revision' folder to locate your manuscript file.

Please include the following items when submitting your revised manuscript:

* A letter that responds to each point raised by the editor and reviewer(s). You should upload this letter as a separate file labeled 'Response to Reviewers'. This file does not need to include responses to any formatting updates and technical items listed in the 'Journal Requirements' section below.

* A marked-up copy of your manuscript that highlights changes made to the original version. You should upload this as a separate file labeled 'Revised Manuscript with Track Changes'.

* An unmarked version of your revised paper without tracked changes. You should upload this as a separate file labeled 'Manuscript'.

If you would like to make changes to your financial disclosure, competing interests statement, or data availability statement, please make these updates within the submission form at the time of resubmission. Guidelines for resubmitting your figure files are available below the reviewer comments at the end of this letter.

We look forward to receiving your revised manuscript.

Kind regards,

Sophie Helaine

Academic Editor

PLOS Pathogens

Eva Heinz

Section Editor

PLOS Pathogens

Sumita Bhaduri-McIntosh

Editor-in-Chief

PLOS Pathogens

orcid.org/0000-0003-2946-9497

Michael Malim

Editor-in-Chief

PLOS Pathogens

orcid.org/0000-0002-7699-2064

**Additional Editor Comments:**

The manuscript has been reviewed by two experts. One has significant concerns that need to be addressed before we can consider publication of the work.

**Journal Requirements:**

1) Please ensure that the CRediT author contributions listed for every co-author are completed accurately and in full.

At this stage, the following Authors/Authors require contributions: Timesh Pillay, Briac Lemetais, Jessica Huber, Ines Diaz del Olmo, Daniel Zhang, Yan Li, Laura Masino, Sarah Maslen, Mei Liu, Diego Esposito, Jay Hinton, Xiujun Yu, Teresa Thurston, and Katrin Rittinger. Please ensure that the full contributions of each author are acknowledged in the "Add/Edit/Remove Authors" section of our submission form.

The list of CRediT author contributions may be found here: https://journals.plos.org/plospathogens/s/authorship#loc-author-contributions

2) We do not publish any copyright or trademark symbols that usually accompany proprietary names, eg ©,  ®, or TM  (e.g. next to drug or reagent names). Therefore please remove all instances of trademark/copyright symbols throughout the text, including:

- ® on page: 36.

- TM on page: 36..

3) Please upload all main figures as separate Figure files in .tif or .eps format. For more information about how to convert and format your figure files please see our guidelines:

https://journals.plos.org/plospathogens/s/figures

**Reviewers' Comments:**

Reviewer's Responses to Questions

**Part I - Summary**

Please use this section to discuss strengths/weaknesses of study, novelty/significance, general execution and scholarship.

Reviewer #1: This study represents an in depth and extensive interrogation of the activity of the important Salmonella effector kinase SteC. The work appears to be of the highest technical quality and the canvasing of Salmonella SteC sequence variation and evolution adds significantly to the story presented. There are a few things that need fixing, which I describe below.

Reviewer #2: In this manuscript, Pillay and colleagues investigate the kinase activity of the Salmonella effector SteC, which drives actin polymerisation around intracellular Salmonella microcolonies. The authors report that SteC is phosphorylated on residue S379 by a host kinase and that this phosphorylation enhances SteC kinase activity and promotes actin polymerisation.

The central conceptual advance of the manuscript is the identification of S379 as a regulatory residue required for SteC function. It has been known since the original 2008 study that SteC is a kinase, that it induces actin polymerisation, and that its C-terminal region is important for function. Therefore, the novelty of the present study rests primarily on the identification and functional characterisation of S379.

The data convincingly demonstrate that S379 is required for SteC activity, through both biochemical and functional infection assays. However the conclusion that phosphorylation at this site is mediated by a host kinase is less convincingly supported

**********

**Part II – Major Issues: Key Experiments Required for Acceptance**

Please use this section to detail the key new experiments or modifications of existing experiments that should be absolutely required to validate study conclusions.

Generally, there should be no more than 3 such required experiments or major modifications for a "Major Revision" recommendation. If more than 3 experiments are necessary to validate the study conclusions, then you are encouraged to recommend "Reject".

Reviewer #1: Assuming the affinity of mant-AMPPNP equals the affinity of ATP, a difference of ~2-fold (5.4 and 11.8 μM) cannot be of biological/biochemical significance. Concentrations of ATP in cells are around 1 millimolar, and such tiny difference in affinity won't produce any meaningful change in "activation". The authors must remove that conclusion from the paper, specifically page 11 lines 247-249. Their data suggests the opposite of what the authors concluded, that the S379D mutation in fact has only minimal effect on SteC activity.

Reviewer #2: -There is inconsistency in assay conditions, which may have been contributed to some confusion on my end in interpreting the results. But in general different SteC constructs are used (full length, 210–457, 210–429, 200–377) and it isn’t clearly explained why. It isn’t always clear which purification system has been used (E. coli versus insect cells). In addition, kinase assay conditions differ (e.g., substrate concentrations of 5 µM versus 100 µM; ATP inclusion or omission are not always clearly explained).

-As previously demonstrated, and also shown here (Fig. 1B), SteC undergoes autophosphorylation when purified from E. coli. It is therefore unclear how the authors definitively distinguish autophosphorylation from host-kinase-mediated phosphorylation. What is the identity of the autophosphorylation site observed in Fig. 1B? Is this site S379?If SteC purified from E. coli is capable of autophosphorylation and activity, how important is host kinase phosphorylation?

-In Fig. 2B, the quantification of S379 phosphorylation across expression systems is difficult to interpret. It is not clear that all primary data are shown, and the explanation in the text is somewhat confusing.

-2C shows that SteC phosphorylation of its substrate is increased when SteC is purified from insect cells, but this experiment doesn’t include the SteC phosphorylation by autoradiography, so it is difficult to see how much insect cell purification contributes to SteC phosphorylation. Here the S379 mutation is not tested. S379 is tested in 2E but this is confusing because in that experiment SteC is purified from E. coli, therefore showing that S379 phosphorylation is not in this context mediated by a host kinase? Why is SteC not autophosphorylated in these conditions (when it was in 1B)?

-Fig. 2H does suggests that murine cell lysate can phosphorylate SteC K256H, consistent with host-mediated phosphorylation. However, I find the experiment confusing and somewhat inconsistent with the rest of the figure: Why is FMNL1 included in the control condition?Why is a different SteC construct (210–429) used here compared to other assays? Why does SteC not appear to autophosphorylate in this assay (left)?

-Most importantly, if SteC activation is indeed mediated by a host kinase, this kinase should be identified or at least narrowed down. At present, no kinase inhibitors or genetic knockouts are tested, no consensus motif analysis is presented, no candidate kinases are proposed, no infection-based phospho-analysis is shown.

Given that host-mediated activation is the central conceptual advance of the manuscript, the lack of identification of the responsible kinase (and how it affects Salmonella infection/actin polymerisation) substantially limits impact.

-Finally, the discussion of the C-terminal tail is unclear and seems disconnected from the rest of the study. The discrepancy between in vitro activity and cellular phenotype is intriguing but insufficiently explained. The mechanistic role of the C-tail during infection remains unresolved and somewhat obscures the central narrative.

**********

**Part III – Minor Issues: Editorial and Data Presentation Modifications**

Please use this section for editorial suggestions as well as relatively minor modifications of existing data that would enhance clarity.

Reviewer #1: Figures can be tightened up. For example, Fig. 1 D has the legend repeated three times and the units of protein concentration (mg/mL) repeated 18 times.

"K" in "Kd" must be italicized.

Reviewer #2: (No Response)

**********

PLOS authors have the option to publish the peer review history of their article (what does this mean?). If published, this will include your full peer review and any attached files.

If you choose “no”, your identity will remain anonymous but your review may still be made public.

**Do you want your identity to be public for this peer review?**  For information about this choice, including consent withdrawal, please see our Privacy Policy.

Reviewer #1: No

Reviewer #2: No

[NOTE: If reviewer comments were submitted as an attachment file, they will be attached to this email and accessible via the submission site. Please log into your account, locate the manuscript record, and check for the action link "View Attachments". If this link does not appear, there are no attachment files.]

**Figure resubmission:**

-->While revising your submission, we strongly recommend that you use PLOS’s NAAS tool (https://ngplosjournals.pagemajik.ai/artanalysis) to test your figure files. NAAS can convert your figure files to the TIFF file type and meet basic requirements (such as print size, resolution), or provide you with a report on issues that do not meet our requirements and that NAAS cannot fix.

After uploading your figures to PLOS’s NAAS tool - https://ngplosjournals.pagemajik.ai/artanalysis, NAAS will process the files provided and display the results in the "Uploaded Files" section of the page as the processing is complete. If the uploaded figures meet our requirements (or NAAS is able to fix the files to meet our requirements), the figure will be marked as "fixed" above. If NAAS is unable to fix the files, a red "failed" label will appear above. When NAAS has confirmed that the figure files meet our requirements, please download the file via the download option, and include these NAAS processed figure files when submitting your revised manuscript.

**Reproducibility:**

To enhance the reproducibility of your results, we recommend that authors of applicable studies deposit laboratory protocols in protocols.io, where a protocol can be assigned its own identifier (DOI) such that it can be cited independently in the future. Additionally, PLOS ONE offers an option to publish peer-reviewed clinical study protocols. Read more information on sharing protocols at https://plos.org/protocols?utm_medium=editorial-email&utm_source=authorletters&utm_campaign=protocols

# Author comment: Author response to Decision Letter 1

**DOI:** 10.1371/journal.ppat.1014424.r002

31 Mar 2026

Attachment Submitted filename: Response to reviewers.pdf

# Peer review: Decision Letter 1

**DOI:** 10.1371/journal.ppat.1014424.r003

**Authors:** Sophie Helaine, Eva Heinz

10 May 2026

-->PPATHOGENS-D-26-00083R1

Salmonella effector kinase SteC is activated by host-mediated phosphorylation

PLOS Pathogens

Dear Dr. Rittinger,

Thank you for submitting your manuscript to PLOS Pathogens. After careful consideration, we feel that it has merit but does not fully meet PLOS Pathogens's publication criteria as it currently stands. Therefore, we invite you to submit a revised version of the manuscript that addresses the points raised during the review process.

Please submit your revised manuscript by Jul 09 2026 11:59PM. If you will need more time than this to complete your revisions, please reply to this message or contact the journal office at plospathogens@plos.org. When you're ready to submit your revision, log on to https://www.editorialmanager.com/ppathogens/ and select the 'Submissions Needing Revision' folder to locate your manuscript file.

Please include the following items when submitting your revised manuscript:

* A letter that responds to each point raised by the editor and reviewer(s). You should upload this letter as a separate file labeled 'Response to Reviewers'. This file does not need to include responses to any formatting updates and technical items listed in the 'Journal Requirements' section below.

* A marked-up copy of your manuscript that highlights changes made to the original version. You should upload this as a separate file labeled 'Revised Manuscript with Track Changes'.

* An unmarked version of your revised paper without tracked changes. You should upload this as a separate file labeled 'Manuscript'.

If you would like to make changes to your financial disclosure, competing interests statement, or data availability statement, please make these updates within the submission form at the time of resubmission. Guidelines for resubmitting your figure files are available below the reviewer comments at the end of this letter.

We look forward to receiving your revised manuscript.

Kind regards,

Sophie Helaine

Academic Editor

PLOS Pathogens

Eva Heinz

Section Editor

PLOS Pathogens

Sumita Bhaduri-McIntosh

Editor-in-Chief

PLOS Pathogens

orcid.org/0000-0003-2946-9497

Michael Malim

Editor-in-Chief

PLOS Pathogens

orcid.org/0000-0002-7699-2064

**Additional Editor Comments:**

One reviewer highlights that some of their comments remain unanswered, and the paper's conclusions and title have not been edited accordingly. I would like to give you another opportunity to thoroughly address their comments. If you deemed it impossible to answer, your best next move would be to start again elsewhere at this point, as it is unlikely that the reviewer will change their mind.

**Journal Requirements:**

If the reviewer comments include a recommendation to cite specific previously published works, please review and evaluate these publications to determine whether they are relevant and should be cited. There is no requirement to cite these works unless the editor has indicated otherwise.

1) In the online submission form, you indicated that your data will be submitted to a repository upon acceptance. We strongly recommend all authors deposit their data before acceptance, as the process can be lengthy and hold up publication timelines. Please note that, though access restrictions are acceptable now, your entire minimal dataset will need to be made freely accessible if your manuscript is accepted for publication. This policy applies to all data except where public deposition would breach compliance with the protocol approved by your research ethics board. If you are unable to adhere to our open data policy, please kindly revise your statement to explain your reasoning and we will seek the editor's input on an exemption.

**Reviewers' Comments:**

Reviewer's Responses to Questions

**Part I - Summary**

Please use this section to discuss strengths/weaknesses of study, novelty/significance, general execution and scholarship.

Reviewer #1: The authors have carefully and successfully addressed my concerns.

Reviewer #2: I appreciate the authors’ efforts to clarify several of the points raised in the initial review, and the revised manuscript is clearer in parts. However, while some of the inconsistencies are discussed, they are not fully resolved. Moreover, the central conclusion and title of the study are that SteC is phosphorylated by a host kinase. While the data support host-mediated phosphorylation, they also indicate that autophosphorylation at S379 occurs at levels that are at least partially comparable, and this is not fully reconciled with the overall interpretation.

**********

**Part II – Major Issues: Key Experiments Required for Acceptance**

Please use this section to detail the key new experiments or modifications of existing experiments that should be absolutely required to validate study conclusions.

Generally, there should be no more than 3 such required experiments or major modifications for a "Major Revision" recommendation. If more than 3 experiments are necessary to validate the study conclusions, then you are encouraged to recommend "Reject".

Reviewer #1: N/A

Reviewer #2: 1-Autophosphorylation versus host-mediated phosphorylation remains unclear

The data show substantial phosphorylation of S379 in SteC purified from E. coli (Fig. 2B). Even if these levels are 50% lower than in insect cells, they are dependent on the kinase activity of SteC. This is not sufficiently acknowledged in the interpretation. While the authors propose that this reflects co-translational autophosphorylation that is largely lost once the protein is folded, this explanation is not experimentally demonstrated and remains difficult to reconcile with the in vitro kinase assays.

As a result, the conclusion that S379 phosphorylation is predominantly host-mediated remains insufficiently supported. For example, Fig. 2E is used to demonstrate that S379 phosphorylation is essential for SteC activity against FMNL1, yet SteC is purified from E. coli in this context. This raises the question of how essential host kinase activity is for SteC function (This point was raised in the original review but was not addressed).

Given the importance of distinguishing host- versus auto-phosphorylation for the conclusions of this study, autoradiography for SteC phosphorylation should be consistently shown across all relevant experiments.

2- Inconsistency in constructs, systems, and assay conditions

While the authors have provided explanations for the use of different constructs, expression systems, and assay conditions, these inconsistencies remain. The explanations improve clarity but do not resolve the underlying issues.

For example, in Fig. 2B, the key comparison between SteC purified from E. coli and insect cells examines the proportion of phosphorylated protein, yet this comparison is not performed using the same SteC constructs. There may be valid reasons for this, but these are not clearly explained, making it difficult to interpret the comparison and assess whether the observed differences reflect biological effects or differences in constructs.

3- Host kinase claim remains insufficiently supported

While finding the specific upstream host kinase might be outside the scope of this study, the paper strongly emphasizes that host kinases activate SteC. Without identifying the kinase or doing functional validation (like testing inhibitor sensitivity, testing candidates), this part of the model isn't well supported.

4- C-terminal tail section remains unclear

The discussion of the C-terminal tail remains insufficiently integrated with the rest of the study. While the discrepancy between in vitro activity and cellular phenotype is noted, the mechanistic role of the C-tail during infection remains unresolved and somewhat obscures the central narrative.

**********

**Part III – Minor Issues: Editorial and Data Presentation Modifications**

Please use this section for editorial suggestions as well as relatively minor modifications of existing data that would enhance clarity.

Reviewer #1: N/A

Reviewer #2: (No Response)

**********

PLOS authors have the option to publish the peer review history of their article (what does this mean?). If published, this will include your full peer review and any attached files.

If you choose “no”, your identity will remain anonymous but your review may still be made public.

**Do you want your identity to be public for this peer review?**  For information about this choice, including consent withdrawal, please see our Privacy Policy.

Reviewer #1: No

Reviewer #2: No

**Figure resubmission:**

-->While revising your submission, we strongly recommend that you use PLOS’s NAAS tool (https://ngplosjournals.pagemajik.ai/artanalysis) to test your figure files. NAAS can convert your figure files to the TIFF file type and meet basic requirements (such as print size, resolution), or provide you with a report on issues that do not meet our requirements and that NAAS cannot fix.

After uploading your figures to PLOS’s NAAS tool - https://ngplosjournals.pagemajik.ai/artanalysis, NAAS will process the files provided and display the results in the "Uploaded Files" section of the page as the processing is complete. If the uploaded figures meet our requirements (or NAAS is able to fix the files to meet our requirements), the figure will be marked as "fixed" above. If NAAS is unable to fix the files, a red "failed" label will appear above. When NAAS has confirmed that the figure files meet our requirements, please download the file via the download option, and include these NAAS processed figure files when submitting your revised manuscript.

**Reproducibility:**

To enhance the reproducibility of your results, we recommend that authors of applicable studies deposit laboratory protocols in protocols.io, where a protocol can be assigned its own identifier (DOI) such that it can be cited independently in the future. Additionally, PLOS ONE offers an option to publish peer-reviewed clinical study protocols. Read more information on sharing protocols at https://plos.org/protocols?utm_medium=editorial-email&utm_source=authorletters&utm_campaign=protocols

# Author comment: Author response to Decision Letter 2

**DOI:** 10.1371/journal.ppat.1014424.r004

15 Jun 2026

Attachment Submitted filename: Response_to_Reviewers_Round2.pdf

# Editor report: Decision Letter 2

**DOI:** 10.1371/journal.ppat.1014424.r005

**Authors:** Sophie Helaine, Eva Heinz

30 Jun 2026

Dear Dr Rittinger,

We are pleased to inform you that your manuscript 'Salmonella effector kinase SteC is activated by phosphorylation at Serine 379' has been provisionally accepted for publication in PLOS Pathogens.

Before your manuscript can be formally accepted you will need to complete some formatting changes, which you will receive in a follow up email. A member of our team will be in touch with a set of requests.

Please note that your manuscript will not be scheduled for publication until you have made the required changes, so a swift response is appreciated.

IMPORTANT: The editorial review process is now complete. PLOS will only permit corrections to spelling, formatting or significant scientific errors from this point onwards. Requests for major changes, or any which affect the scientific understanding of your work, will cause delays to the publication date of your manuscript.

Should you, your institution's press office or the journal office choose to press release your paper, you will automatically be opted out of early publication. We ask that you notify us now if you or your institution is planning to press release the article. All press must be co-ordinated with PLOS.

Thank you again for supporting Open Access publishing; we are looking forward to publishing your work in PLOS Pathogens.

Best regards,

Sophie Helaine

Academic Editor

PLOS Pathogens

Eva Heinz

Section Editor

PLOS Pathogens

Sumita Bhaduri-McIntosh

Editor-in-Chief

PLOS Pathogens

orcid.org/0000-0003-2946-9497

Michael Malim

Editor-in-Chief

PLOS Pathogens

orcid.org/0000-0002-7699-2064

***********************************************************

Reviewer Comments (if any, and for reference):

# Editor report: Acceptance letter

**DOI:** 10.1371/journal.ppat.1014424.r006

**Authors:** Sophie Helaine, Eva Heinz

Dear Dr Rittinger,

We are delighted to inform you that your manuscript, "Salmonella effector kinase SteC is activated by phosphorylation at Serine 379," has been formally accepted for publication in PLOS Pathogens.

We have now passed your article onto the PLOS Production Department who will complete the rest of the pre-publication process. All authors will receive a confirmation email upon publication.

The corresponding author will soon be receiving a typeset proof for review, to ensure errors have not been introduced during production. Please review the PDF proof of your manuscript carefully, as this is the last chance to correct any scientific or type-setting errors. Please note that major changes, or those which affect the scientific understanding of the work, will likely cause delays to the publication date of your manuscript. Note: Proofs for Front Matter articles (Pearls, Reviews, Opinions, etc...) are generated on a different schedule and may not be made available as quickly.

Soon after your final files are uploaded, the early version of your manuscript, if you opted to have an early version of your article, will be published online. The date of the early version will be your article's publication date. The final article will be published to the same URL, and all versions of the paper will be accessible to readers.

For Research Articles, you will receive an invoice from PLOS for your publication fee after your manuscript has reached the completed accept phase. If you receive an email requesting payment before acceptance or for any other service, this may be a phishing scheme. Learn how to identify phishing emails and protect your accounts at https://explore.plos.org/phishing.

Thank you again for supporting open-access publishing; we are looking forward to publishing your work in PLOS Pathogens.

Best regards,

Sumita Bhaduri-McIntosh

Editor-in-Chief

PLOS Pathogens

orcid.org/0000-0003-2946-9497

Michael Malim

Editor-in-Chief

PLOS Pathogens

orcid.org/0000-0002-7699-2064
